# Paradigm shift in obesity treatment: an extensive review of current pipeline agents

**DOI:** 10.55730/1300-0144.5938

**Published:** 2025-01-15

**Authors:** Ecesu ÇETİN, Brian PEDERSEN, Mehmet Furkan BURAK

**Affiliations:** 1 Division of Endocrinology, Diabetes and Hypertension, Brigham and Women’s Hospital and Harvard Medical School, Boston, MA, United States; 2 Department of Molecular Metabolism, Harvard T.H. Chan School of Public Health, Boston, MA, United States

**Keywords:** Obesity, glucagon-like peptide 1, antiobesity drugs, metabolic syndrome, overweight

## Abstract

Obesity is a multifaceted disease that poses a significant public health challenge. Recent discoveries in understanding the biological pathways that regulate satiety and metabolism have led to a shift in the treatment paradigm for obesity. Thus, the gap between pharmacological and surgical interventions has diminished. The latest approved antiobesity medications help to achieve weight loss comparable to surgery. These GLP-1 analog-based therapies not only cause substantial weight loss but also improve obesity-associated comorbidities. However, there are still unmet needs in obesity care, and treatment options with alternative pathways are necessary. Whether achieved through lifestyle changes or medication, weight loss often leads to muscle mass loss and reduced energy expenditure, resulting in rebound weight gain. Moreover, addressing severe obesity and comorbidities, such as metabolic-associated fatty liver disease (MAFLD), metabolic dysfunction-associated steatohepatitis (MASH), heart failure with preserved ejection fraction, and obstructive sleep apnea, necessitates the development of additional therapeutic strategies. Various antiobesity medications with novel mechanisms of action are currently in the pipeline. Myostatin-activin pathway inhibitors are under development to preserve muscle mass, and combination therapies with glucagon agonists address MAFLD and MASH. Amylin agonists offer a promising alternative to those unable to tolerate GLP-1 analogs. Mitochondrial uncouplers are under investigation for enhancing energy expenditure, NLRP-3 inhibitors for reducing inflammation, and GWAS targets for additional weight loss benefits. Combination therapies, such as dual or triple hormonal receptor agonists, are being developed to maximize weight loss and optimize tolerability. These emerging medications in the clinical trial pipeline show promise for more tolerable and sustainable obesity management.

## 1. Introduction

Obesity is a complex disease and a public health challenge resulting from dysregulated central body weight control [1,2]. Complex regulatory mechanisms govern body weight with a preference for defending fat mass. However, this mechanism presents a challenge in the modern era, as it protects abnormally elevated body weights, hindering sustainable efforts for fat loss. A net negative energy balance activates feedback loops in the brain, leading to weight regain. Current therapeutic strategies for addressing obesity mainly aim to decrease the physiological inclination in energy regulation by modulating eating behavior [1]. Recent findings on pathways in the gastrointestinal tract that impact satiety and the interactions between peripheral metabolic and central nervous system elements have led to a transformational paradigm shift in obesity treatment [1,2]. Prior weight management strategies to increase basal energy expenditure, such as administering excess thyroid hormone or dinitrophenol, have been hampered by significant cardiovascular safety concerns, precluding their application in clinical pharmacology [1]. Rather than chemical compounds with numerous side effects, scientists have identified regulatory signals from the gastrointestinal tract and adipose tissue, which interact with specific regions in the central nervous system, controlling energy homeostasis and hunger or satiety perception. Therefore, restoring the physiology with nutrient-stimulated hormones yields significant benefits [1,2]. With the advent of second-generation antiobesity medications, however, there is a notable enhancement in weight loss outcomes. For example, tirzepatide, in combination with lifestyle interventions, results in 26.6% weight loss at week 72 [3]. In comparison, surgical interventions typically result in a 25%–33% weight reduction [1,4]. Hence, the therapeutic gap between medical and surgical methodologies for managing obesity is diminishing. Obesity often appears alongside a group of interconnected immunometabolic disorders such as type 2 diabetes and fatty liver disease. Innovative antiobesity medications now enable effective treatment of both obesity and its related conditions [1]. Neuronal networks influence food intake via different pathways. Thus, the efficacy of targeting a single pathway or receptor for weight loss is limited. Therefore, ongoing studies have investigated combination therapies, such as dual or triple hormonal receptor agonists, for potentiated effects and enhanced tolerability [1,5]. Table 1 displays currently approved antiobesity medications by the U.S. Food and Drug Administration (FDA), and Table 2 provides an overview of pipeline agents undergoing evaluation in preclinical and clinical studies.

## 2. Current FDA-approved antiobesity medications

### 2.1. Phentermine/topiramate

Phentermine and topiramate (PHEN/TPM) combination therapy is approved for weight management in adults as well as pediatric patients aged 12 years and older with obesity [6]. Phentermine hydrochloride is a sympathomimetic amine that suppresses appetite [7,8]. It was approved for short-term management of obesity by the FDA in 1959 [7]. Following its approval, it remained the most commonly prescribed antiobesity medication in the U.S. [9]. Topiramate is an antiepileptic medication that promotes significant weight loss effects, potentially through higher energy expenditure and lower caloric intake. However, the adverse effects at optimal doses limit its usage in weight management as a monotherapy [7]. PHEN/TPM is the combination therapy of phentermine and topiramate in an oral formulation. Its FDA approval for managing obesity was primarily supported by three clinical trials (EQUIP, CONQUER, and SEQUEL). Across these Phase 3 trials, treatment with PHEN/TPM consistently resulted in statistically significant weight reduction compared to placebo [8]. However, PHEN/TPM is linked to significant side effects, including tachycardia, suicidal ideation, and growth restriction in adolescents. As a teratogenic drug, it poses a high risk of birth defects, such as cleft lip and palate, necessitating pregnancy testing and effective contraception for women of childbearing age. Due to these risks, access to this medication is restricted to the Risk Evaluation and Mitigation Strategy program by the FDA [6].

### 2.2. Bupropion/naltrexone

Bupropion/naltrexone combination therapy was approved by the FDA in 2014 for long-term obesity management [10]. Bupropion is a dopamine/norepinephrine reuptake inhibitor that activates hypothalamic proopiomelanocortin (POMC) neurons, leading to subsequent effects that decrease food consumption and enhance energy expenditure [10,11]. Naltrexone is an opioid receptor antagonist that inhibits the self-inhibition of POMC neurons through opioid receptors and enhances POMC activity synergistically [11]. Bupropion/naltrexone is contraindicated in patients with uncontrolled hypertension, and regular monitoring of blood pressure is advised during treatment. Additionally, due to the bupropion component, it reduces the threshold for seizures and is contraindicated in patients with a seizure history, a history of anorexia/bulimia nervosa, or opioid usage. It has a black box warning due to increased suicidality risk. Additionally, its usage in pregnancy is contraindicated [10].

### 2.3. Orlistat

Orlistat is an irreversible pancreatic and gastric lipase inhibitor approved by the FDA for weight management in 1999. It inhibits the breakdown of triglycerides into free fatty acids and monoglycerides, reducing dietary fat absorption and promoting a caloric deficit [12]. However, it is not widely used due to its minimal ability to moderate weight loss and excessive gastrointestinal side effects.

### 2.4. MC4 receptor agonist: setmelanotide

The melanocortin-4 receptor (MC4R) pathway is important in coordinating satiety and energy consumption [1]. In a nourished state, leptin physiologically activates its receptor (LEPR), triggering POMC neurons to secrete melanocyte-stimulating hormone (MSH) via the enzyme proprotein convertase 1/3, which is encoded by the proprotein convertase subtilisin/kexin type 1 (PCSK1) gene. Afterward, MSH stimulates the MC4Rs located in melanocortin neurons within the paraventricular nucleus [1], leading to reduced appetite, increased sympathetic activity, and enhanced energy expenditure [1,13]. The genetic variants or mutations within the MC4R pathway can disrupt the regulation of hunger and energy expenditure. As a result, individuals with these genetic variants may experience severe, early-onset obesity characterized by hyperphagia [13,14]. Thus, MC4R agonism is being investigated as a potential treatment for weight control. Setmelanotide is an FDA-approved agonist of MC4R for adults and children aged 6 years and older diagnosed with monogenic obesity linked to specific unbenign genetic variants of POMC, PCSK1, or LEPR or those with Bardet-Biedl syndrome [13,15]. It imitates the MSH and recovers the defective signaling in the MC4R pathway by restoring the downstream mechanism. By doing so, setmelanotide leads to decreased body weight through modulating appetite and energy consumption. A 66-week international clinical trial of setmelanotide in individuals with obesity due to Bardet-Biedl syndrome demonstrated more than a 10% reduction in body weight in 32.3% of participants by the end of the trial [13]. Moreover, setmelanotide has shown potential as a treatment for hypothalamic obesity, a rare condition resulting from damage to the hypothalamus, such as from craniopharyngiomas, other tumors located in the suprasellar region, or the treatment for these tumors, which can disrupt the MC4R pathway. There are no approved treatments available for hypothalamic obesity. However, setmelanotide, currently in clinical trials, shows promise as a potential therapy for this condition. In a phase 2 trial for acquired hypothalamic obesity, participants experienced an average body mass index (BMI) decrease of 15% at week 16 and 26% at 12 months with setmelanotide [14]. Examinations on the efficacy of current FDA-approved medications for hypothalamic obesity will be carried out in the future. The literature has reported the weight loss effects of GLP-1-based therapies for individuals with obesity due to MC4R pathway mutations [16], and GLP-1 agonists might be an effective option in managing hypothalamic obesity.

### 2.5. GLP-1 agonists and GIP/GLP-1 dual agonists

Glucagon-like peptide 1 (GLP-1) is a peptide hormone synthesized within the endocrine L-cells of the intestinal epithelium [17]. In addition, it acts as a neurotransmitter in the brainstem, nucleus tractus solitarius, and hypothalamus and exerts specific effects across various organ systems [1,17]. It decreases gluconeogenesis and steatosis in the liver, slows gastric emptying, reduces gastrointestinal motility in the intestine, reduces food intake, modifies reward behavior, influences palatability in the brain, enhances insulin sensitivity, promotes glucose uptake in muscle, boosts insulin secretion, supports insulin synthesis and beta cell survival, diminishes apoptosis, and limits glucagon secretion in the pancreas [18]. All of these effects synergistically contribute to achieving weight loss. GLP-1 itself, while effective, has limitations as a therapeutic method due to its highly brief half-life (approximately 2 min) and rapid renal elimination owing to its high hydrophilicity. These challenges were overcome by creating GLP-1 receptor agonists (GLP-1 RAs), which are resistant to degradation and renal clearance [19]. The duration of stimulation can significantly influence appetite and food intake due to the complex regulatory pathways involved. For instance, long-acting semaglutide demonstrates greater potency and enhanced central uptake than short-acting liraglutide [1]. Semaglutide and tirzepatide are prominent among current long-acting drugs and exemplify this innovative approach. Of note, in rodent studies, GLP-1 analogs have been associated with the development of thyroid C-cell tumors, including adenomas and carcinomas, in a dose-dependent and treatment-duration-dependent manner at exposure levels relevant to clinical use. However, it is essential to underline whether GLP-1 receptor agonists have a similar effect in humans, which remains unclear. Despite extensive investigations, no study shows a causal relationship in humans. GLP-1 RAs are contraindicated^1^ only for individuals with a medullary thyroid carcinoma history (personal/familial) or those with Multiple Endocrine Neoplasia syndrome 2.

#### 2.5.1. Liraglutide

The FDA approved liraglutide as the first daily injectable GLP-1 RA to treat diabetes. Considering the significant rise in obesity rates, studies have focused on assessing the impact of liraglutide on body weight and its tolerability in individuals without diabetes [19]. In a 56-week trial with 3731 nondiabetic patients with obesity, those receiving liraglutide lost an average of 8.4 ± 7.3 kg, whereas those on placebo lost 2.8 ± 6.5 kg. Compared to 27.1% in the placebo group, 63.2% of the liraglutide group achieved at least a 5% reduction in body weight. Overall, a 3.0-mg subcutaneous injection of liraglutide is linked to reduced body weight and improved metabolic control [20].

#### 2.5.2. Semaglutide

Semaglutide is a GLP-1 receptor agonist administered via a once-weekly subcutaneous injection [2]. Initially approved by the FDA for the treatment of type 2 diabetes in 2017, it subsequently received FDA approval for treating obesity in adults and pediatric patients above the age of 12.^1^ In a large-scale, multinational, randomized trial spanning 129 sites across 16 countries, conducted with 1961 adults with a BMI of 30 or higher, without diabetes, by week 68, the semaglutide group exhibited a substantial mean reduction in body weight (−14.9%) compared to the placebo group (−2.4%). One-third of the participants who were administered semaglutide achieved a weight loss of at least 20% from their initial weight, a reduction like that observed in patients 1 to 3 years postbariatric surgery. The semaglutide group also experienced a greater absolute reduction in body weight from baseline to week 68 (−15.3 kg) compared to placebo (−2.6 kg). In addition, significant improvements were observed in cardiometabolic measures with semaglutide, including blood pressure, glycated hemoglobin levels, and lipid profiles [2].

#### 2.5.3. Tirzepatide

Tirzepatide is a synthetic peptide that acts as a dual agonist at the glucose-dependent insulinotropic polypeptide (GIP) and GLP-1 receptors. By integrating signals from both GIP and GLP-1 receptor pathways in the brain, tirzepatide has the potential to achieve greater weight reduction compared to selective GLP-1 receptor agonists alone. GIP agonism provides additional benefits such as improved adipose tissue health, reduced accumulation of ectopic fat, and enhanced tolerability with an attenuation of gastrointestinal side effects [1]. Tirzepatide was approved by the FDA for type 2 diabetes in 2022 and subsequently received FDA approval for weight management in 2023 [21]. The SURMOUNT clinical trials are phase-3 multicenter studies investigating the safety and efficacy of once weekly tirzepatide subcutaneous injections versus placebo in participants with obesity. SURMOUNT-1 and SURMOUNT-2 focused on fixed-dose efficacy and safety studies, while SURMOUNT-3 and SURMOUNT-4 investigated clinically relevant maximum tolerated doses [21]. In the SURMOUNT-1 trial conducted with 2539 adults, an average weight loss of −20.9% occurred in the 15-mg dose group at week 72 compared to −3.1% with placebo. Additionally, improvements were present in all predefined cardiometabolic parameters, including systolic and diastolic blood pressure, fasting insulin levels, and lipid profiles [22]. In the SURMOUNT-3 trial, tirzepatide showed a clinically significant additional decrease in body weight among overweight or obese patients after integrating intensive lifestyle interventions, resulting in a remarkable 26.6% weight loss at week 72 [3].

The predominant side effects of GLP-1 agonists are gastrointestinal, including transient symptoms such as nausea, vomiting, or diarrhea. Because these adverse effects usually occur during dose escalation periods, the optimal approach to increasing patient tolerance is to slowly titrate the medication [2,22].

#### 2.5.4. Insights into obesity comorbidities from FDA-approved antiobesity medications

FDA-approved antiobesity medications have been evaluated for their effects on comorbidities. These clinical trial outcomes provide important insights into their broader therapeutic implications.

Previously, the multinational SUSTAIN-6 trial demonstrated the efficacy of semaglutide in reducing cardiovascular events in 3297 patients with type 2 diabetes over 104 weeks. The administration of semaglutide significantly reduced the rates of cardiovascular mortality, nonfatal myocardial infarction, and stroke compared to the placebo [23]. Even in patients without diabetes, beneficial effects were observed. The SELECT trial investigated semaglutide’s impact on cardiovascular health in 17,604 overweight or obese adults without diabetes but who had a history of myocardial infarction, stroke, and/or peripheral artery disease. The findings demonstrated a significant 20% reduction in the risk of major adverse cardiovascular events among patients treated with semaglutide compared to placebo at an average follow-up period of 39.8 months. This clinical trial demonstrated that semaglutide lowers cardiovascular risks in overweight individuals with established cardiovascular disease [24,25]. In line with these results, the FDA approved the cardiovascular risk reduction indication of semaglutide in March 2024 [26].

Additionally, semaglutide improves kidney outcomes. The SELECT trial demonstrated that once-weekly semaglutide 2.4 mg reduced the incidence of the primary composite renal endpoint, including reduced renal disease mortality, decreased stage 5 chronic kidney disease, and a sustained eGFR decline of ≥50%. The semaglutide group had a significantly lower incidence (1.8%) than the placebo group (2.2%). After 104 weeks, the semaglutide group showed a smaller reduction in eGFR with a difference of 0.75 mL/min/1.73 m^2^ compared to the placebo. For participants with a baseline eGFR below 60 mL/min/1.73 m^2^, the benefit was even more pronounced with 2.19 mL/min/1.73 m^2^. This shows a significant improvement in kidney function in patients without diabetes, particularly those with advanced renal impairment at study onset [27]. Furthermore, the FLOW trial, involving 3533 participants with type 2 diabetes and chronic kidney disease, demonstrated that the occurrence of primary renal endpoint decreased by 24% in the semaglutide recipients compared to the placebo, indicating the protective effect of semaglutide on kidney function and overall survival in this high-risk population with diabetes [28]. The SOUL trial will investigate the impact of daily oral semaglutide on the incidence of cardiovascular events in 9650 patients with known atherosclerotic cardiovascular disease/chronic kidney disease and diabetes [29].

Moreover, semaglutide is beneficial for heart failure with preserved ejection fraction (HFpEF), with markedly improved symptoms and enhanced exercise capacity of patients. This is important, considering there are no approved therapies for HFpEF associated with obesity [30]. The Phase 3 ESSENCE trial has evaluated the impact of semaglutide on 1200 patients with metabolic dysfunction-associated steatohepatitis (MASH) and fibrosis.^2^

At present, three ongoing clinical trials are exploring semaglutide’s impact on reducing alcohol consumption, with another trial investigating its effects on nicotine intake.^3^ Tirzepatide is also the subject of several studies investigating its therapeutic potential in comorbidities. The SURMOUNT-MMO trial is a Phase 3 study assessing the impact of tirzepatide on 15,374 participants aged 40 or older with obesity and established cardiovascular disease risk.^4^ The SURMOUNT-OSA study comprised two Phase 3 trials assessing tirzepatide in patients diagnosed with moderate to severe obstructive sleep apnea (OSA) and obesity over 52 weeks. Trial 1 included participants not using positive airway pressure (PAP) therapy, while Trial 2 included those on PAP therapy. Tirzepatide significantly reduced the apnea-hypopnea index, with an average decrease of 20.0 events per hour in Trial 1 and 23.8 events per hour in Trial 2 compared to placebo. Improvements were also noted in hypoxia, hs-CRP levels, and blood pressure. These findings are particularly significant considering the causal association between obesity and OSA and the recognized role of OSA as a risk factor for cardiovascular complications [31]. Additionally, a Phase 2 trial evaluating tirzepatide for MASH treatment revealed that the percentage of participants achieving MASH resolution without any worsening of fibrosis was 62% in the 15-mg tirzepatide group, compared to 10% in the placebo group [32]. This investigation is crucial for understanding tirzepatide’s potential as a therapeutic option for patients with MASH, a condition for which effective treatments are currently limited.

## 3. Current unmet needs

I. Out of 813 million people suffering from obesity, approximately 2% have access to medical treatment.^5^ Access to medications remains a significant challenge due to affordability and availability issues. Even in the US, medications such as semaglutide are listed on the FDA’s Drug Shortages list due to high demand and limited supply.^6^ Addressing these issues is crucial to ensuring that effective obesity treatments are accessible to everyone, regardless of economic or geographical constraints.

II. An oral form of antiobesity medications is needed, considering shortages in autoinjectors, manufacturing problems with injectables, and patient preferences.

III. GLP-1 agonists, such as semaglutide and tirzepatide, mainly cause gastrointestinal side effects [2,22]. A subset of patients exist who are intolerant to GLP-1 agonists. Although adherence to newer-generation antiobesity medications is higher compared to older-generation ones, only 40% of patients remained on semaglutide treatment at the end of one year [33]. Therefore, an individual’s intolerance to GLP-1 requires treatment through alternative pathways.

IV. Even modest reductions in weight (~7%) can result in muscle mass loss [34]. Currently, no treatment exists that can selectively target fat mass without concurrently causing loss of lean mass. Furthermore, weight loss leads to a decrease in metabolic rate and energy expenditure [35]. Therefore, individuals disadvantageously burn fewer calories for the same level of physical activity, potentially leading to rebound weight regain if medication is discontinued. At the same time, continuing treatment with semaglutide or tirzepatide is required to sustain the achieved weight loss. One year after discontinuing semaglutide, patients regained two-thirds of their initial weight loss, with comparable alterations in cardiometabolic parameters [36]. Similarly, the discontinuation of tirzepatide resulted in significant weight regain. On the other hand, continuing the treatment led to an enhancement in the initial weight loss [37]. Obesity is a chronic disease; thus, continuous treatment is necessary to sustain improvement in weight and overall health, partly due to a decreased metabolic rate. Weight regain and muscle mass maintenance after weight loss remain significant unmet needs in the weight management field.

V. Developing novel medications that enhance energy expenditure safely, rather than merely reducing food intake, is essential. Although muscle mass is one pathway for energy expenditure, some agents can increase energy expenditure through other mechanisms. Dinitrophenol can achieve this by inducing mitochondrial uncoupling; however, its systemic effects pose significant safety concerns [38]. The administration of T3, which also increases basal metabolic rate, was considered a potential weight management strategy [39]. Similarly, mirabegron is an agonist of the β3-adrenergic receptor that has shown effectiveness in activating brown adipose tissue. While animal studies have indicated that mirabegron administration can ameliorate obesity, it has not resulted in significant weight loss in obese patients to date [40]. However, both T3 and mirabegron have been associated with cardiac side effects at therapeutic doses [39,40], and neither is readily available for weight management. In contrast with these two medications, new-generation liver-specific mitochondrial uncouplers, which are in the current pipeline, have a reduced risk of severe adverse effects as they limit systemic exposure.

VI. Current treatment approaches are inadequate in addressing severe obesity, metabolic-associated fatty liver disease (MAFLD), and MASH. Therefore, novel treatment options are required.

## 4. Obesity pipelines

### 4.1. Oral GLP-1 based medications

#### 4.1.1. Oral semaglutide

Semaglutide is also available in an oral formulation and is the first oral peptide GLP-1 agonist approved by the FDA for diabetes management. Ongoing clinical trials are evaluating it for weight management. In a Phase 3 study, 667 participants were randomly allocated to receive either oral semaglutide 50 mg or placebo. The average weight loss at week 68 was 15.1% with oral semaglutide 50 mg compared to 2.4% with placebo, similar to the weight loss observed with the 2.4 mg weekly injectable dose [41]. Despite the high-dose oral formulation demonstrating noninferior efficacy, the impact of food on its absorption presents a disadvantage for chronic disease management.

#### 4.1.2. Orforglipron

Orforglipron is an oral nonpeptide potent partial GLP-1 receptor agonist that potentially reduces receptor desensitization relative to full GLP-1 agonists through a stronger effect on cAMP compared to β-arrestin recruitment. Its pharmacokinetic characteristics allow for once-daily oral dosing. The outcomes of the Phase 2 study showed that orforglipron is noninferior to weekly injectable treatments with a similar adverse event profile [42]. It may also have the advantage of manufacturing ease since it is a nonpeptide small molecule.

#### 4.1.3. GSBR-1290

GSBR-1290 is a once-daily oral, nonpeptide small molecule GLP-1 receptor agonist. The 12-week Phase 2a trial demonstrated an average weight loss of 4.74% after being adjusted for placebo at 56, which indicates comparable early efficacy [43].

#### 4.1.4. CT-996

CT-996 is an oral small molecule GLP-1 receptor agonist administered once daily. Its biased agonism, which selectively activates cAMP signaling, aims to enhance weight reduction and improve tolerability in treating obesity and type 2 diabetes. CT-996 is currently undergoing a Phase 1 trial.^7^

#### 4.1.5. VK2735

VK2735 is a dual GIP and GLP-1 receptor agonist available in subcutaneous and oral formulations. The Phase 2 VENTURE trial, which involved a once-weekly subcutaneous injection, showed a significant average body weight decrease of up to 14.7% at 13 weeks. Moreover, the Phase 1 study, which involved oral VK2735, administered once daily, resulted in positive weight loss and tolerability outcomes [44].

### 4.2. Myostatin-activin pathway inhibitors

Members of the TGF-β superfamily are known to inhibit muscle growth and may exacerbate muscle wasting. These members include activins, growth and differentiation factor-11 (GDF-11), and myostatin (GDF-8). Myostatin, activins, and GDF11 bind to myostatin/activin type II receptor (ActRII) complexes, resulting in purely inhibitory actions within mature myofibers. This interaction stimulates muscle atrophy by reducing protein synthesis, increasing protein degradation, and diminishing signals that promote muscle growth [45]. Myostatin-activin pathway inhibitors, initially developed for patients affected by muscle-wasting diseases such as Duchenne muscular dystrophy, show promise for therapeutic applications to preserve muscle mass while reducing fat tissue during weight loss in obesity treatment [46]. Muscle functions as a key endocrine organ in metabolism. Therefore, inhibiting myostatin and increasing muscle mass is associated with numerous metabolic health benefits, including enhanced basal metabolism and insulin sensitivity, reduced visceral and intramuscular fat, and overall reduction in body fat mass [47]. Because GDF-8 (myostatin) and GDF-11 have 89% sequence identity despite their distinct endogenous functions, selective targeting and inhibition of GDF-8 and minimizing effects on GDF-11 are essential to optimize their therapeutic efficacy [48]. Otherwise, the nonselective inhibition of the ActRII pathway may lead to harmful side effects.

#### 4.2.1. Bimagrumab

Bimagrumab is a human monoclonal antibody that inhibits the ActRII. It inhibits the function of myostatin and other ligands that serve as negative regulators of skeletal muscle. A placebo-controlled, multiple-dose study involving seven treatment groups (Cohorts 1–7) established the viability of weekly subcutaneous administration as an alternative to intravenous dosing for bimagrumab in future clinical trials [49]. Additionally, a Phase 2 randomized clinical trial involving 75 patients with type 2 diabetes and obesity showed that participants in the bimagrumab treatment group experienced significantly greater reductions in total body fat mass (−20.5%), as well as increases in lean mass (3.6%) compared to those receiving placebo over 48 weeks [50]. Recently, nutrient-stimulated hormone-based therapies have been combined with myostatin-activin pathway inhibitors to enhance weight reduction and optimize fat mass loss while preserving muscle mass. A Phase 2b BELIEVE study is currently evaluating bimagrumab both as a monotherapy and in combination with semaglutide for adults who are overweight or obese.^8^

#### 4.2.2. Taldefgrobep alfa (BHV-2000)

Taldefgrobep alfa is a fully human recombinant protein that binds to mature myostatin and the activin receptor type IIB (ActRIIB)–myostatin complex. It was previously tested in clinical trials for Duchenne muscular dystrophy [46]. In preclinical obesity studies, taldefgrobep led to significant, sustained muscle mass improvements and reductions in whole-body fat mass [51].

#### 4.2.3. SRK-439

SRK-439 is a selective myostatin inhibitor designed to treat obesity. Preclinical studies have shown that it promotes healthier weight management by preserving lean muscle mass, reducing fat mass regain after semaglutide discontinuation, and supporting healthy body composition [52].

#### 4.2.4. Trevogrumab and garetosmab

Trevogrumab (REGN 1033) is a fully human monoclonal antibody targeting myostatin (GDF-8) with no measurable GDF-11 affinity [45]. Garetosmab, a fully human monoclonal antibody, is designed to bind and inhibit activin A signaling. In a Phase 1 study, trevogrumab and garetosmab were evaluated in combination and as monotherapy. Common adverse events comprised muscle spasms, myalgia, mouth ulceration, and infections. After administering a single dose of the combination therapy, there was a 7.7% increase in thigh muscle volume by week 8 and a reduction in whole body and android fat mass. The final dose was given in the 12th week. By the 28th week follow-up, abdominal and visceral fat mass was reduced by 14.3% and 20.1%, consecutively, compared to baseline, and it remained sustained over time. Trevogrumab and garetosmab synergistically enhanced muscle and lean mass more than their individual effects and reduced fat mass. Simultaneously inhibiting GDF-8 and activin A could hold promise for obesity management [53]. The Phase 2 COURAGE trial investigates the effects of trevogrumab and garetosmab in conjunction with semaglutide on weight management and fat reduction.^9^

#### 4.2.5. KER-065 and RKER-034

KER-065 is a selective ligand trap of the activin receptor that blocks the effects of myostatin, and activin A. RKER-034 is an investigational molecule that functions similarly to KER-065 by blocking specific TGF-β ligands. Thus, both enhance skeletal muscle mass and promote fat metabolism, significantly increasing lean body mass [54]. A Phase 1 clinical trial is now assessing the safety and tolerability of KER-065 in healthy individuals.^10^

### 4.3. Glucagon addition

Glucagon mainly stimulates the liver to increase hepatic glucose production. Its unique ability to increase energy expenditure in addition to promoting satiety, unlike treatments focused solely on reducing food intake, makes this hormone a promising pharmaceutical option for managing obesity. Importantly, it has potential therapeutic implications for MAFLD and/or MASH because of its capacity to increase hepatic fatty acid oxidation. Indeed, it improves serum lipid profiles and contributes to the reversal of liver steatosis. Accordingly, glucagon is incorporated into combination therapies with GLP-1 agonists [55]. While GLP-1 primarily promotes decreased food intake, glucagon enhances energy expenditure, reduces liver fat, and potentially alleviates MAFLD/MASH.

#### 4.3.1. GIP/GLP-1/Glucagon triple agonist: retatrutide (LY3437943)

Retatrutide is the first triple hormone receptor agonist, activating the GIP, GLP-1, and glucagon receptors. In a Phase 2 trial with 338 adults with obesity, participants were randomly assigned to receive once-weekly subcutaneous doses of retatrutide ranging from 1 mg to 12 mg or placebo over 48 weeks. The 12-mg dose group showed an average weight reduction of 24.2% compared to a decrease of 2.1% in the placebo group [56]. In another Phase 2 trial with 98 participants, the average reduction in liver fat was 82.4% in the 12-mg group, compared to an increase of 0.3% in the placebo group at 24 weeks. Normalization of liver fat (<5%) was attained by 86% in the 12-mg group, while none in the placebo group achieved this outcome. Liver fat reductions were strongly associated with improved metabolic markers indicative of enhanced insulin sensitivity and lipid metabolism [57]. Despite achieving close to surgical-level weight loss and notable reductions in liver fat, it is important to note that there were dose-dependent increases in heart rate peaking at 24 weeks [56]. Retatrutide is currently in the Phase 3 TRIUMPH trial to assess efficacy and safety in individuals with obesity and established cardiovascular disease.^11^

#### 4.3.2. GLP-1 and glucagon dual agonists

##### 4.3.2.1. Survodutide (BI 456906)

Survodutide is a once-weekly injectable dual GLP-1 and glucagon receptor agonist. In a multinational Phase 2 trial across 43 centers involving 386 participants with a BMI ≥27 kg/m^2^ and with no diabetes, participants in the 4.8-mg survodutide group exhibited an average body weight reduction of 14.9% in week 46, compared to a 2.8% decrease in the placebo group [58]. In another Phase 2 clinical trial with 293 participants, 62% of participants in the 4.8-mg dosage group demonstrated improvement in MASH without worsening fibrosis, in contrast to 14% in the placebo group. Moreover, 67% achieved a reduction in liver fat content of at least 30%, and 36% showed improvement in fibrosis by at least one stage [59]. Currently, SYNCHRONIZE Phase 3 trials are investigating survodutide for individuals who are overweight or obese.^12^

##### 4.3.2.2. Pemvidutide

Pemvidutide is an investigational dual GLP-1/glucagon receptor agonist targeting obesity and MASH. The MOMENTUM trial is a 48-week Phase 2 study assessing safety and efficacy in obese and overweight individuals. The average weight loss was 15.6%, and more than 30% of participants experienced weight loss of at least 20% with the 2.4-mg dose. Furthermore, significant decreases were observed in triglycerides (55.8%), total cholesterol (20.0%), and LDL cholesterol (21.8%) concentrations in patients with baseline dyslipidemia. Blood pressure improvements were observed without any clinically significant increases in heart rate or occurrence of arrhythmias [60]. It has received Fast Track designation from the FDA for MASH treatment and is currently under investigation in the Phase 2b IMPACT trial for MASH.^13^

##### 4.3.2.3. Mazdutide (LY3305677)

Mazdutide is a dual GLP-1 and glucagon receptor agonist under development for treating obesity and diabetes. In a Phase 3 clinical trial involving 610 overweight or obese Chinese individuals, the administration of mazdutide led to substantial decreases in body weight and improvements in cardiometabolic risk factors [61].

### 4.4. Amylin agonists

Amylin (AMY) is a pancreatic endocrine hormone cosynthesized with insulin and released by β-cells upon food intake. It stimulates AMY receptors in the nervous system and decreases food consumption. Moreover, AMY improves leptin sensitivity. Leptin is a hormone that suppresses hunger and boosts energy expenditure. Counterintuitively, obesity is characterized by increased leptin production. This is due to leptin resistance. Therefore, improving leptin sensitivity is an important therapeutic target in addressing obesity [62]. However, human AMY has a strong tendency to precipitate and form amyloid fibrils and requires acidic formulation for chemical stability. These challenges have posed a significant obstacle in drug development; however, these barriers were overcome with the discovery that AMY from distinct species does not form amyloid fibrils [63]. Another crucial aspect is the significant homology shared between AMY and calcitonin, both of which interact with heterodimeric receptors in the brain consisting of a core calcitonin receptor (CTR) and receptor activity-modifying proteins (RAMPs) [1,63]. Previously, pramlintide, a specific AMY analog that only binds to RAMP, was developed to treat diabetes mellitus. However, it led to modest weight loss only, which proved inadequate for reversing obesity-related complications. Consequently, new AMY analogs that nonselectively bind to both CTR and RAMPs are being developed. These analogs aim to improve weight control by activating area postrema, nucleus tractus solitarius, dopaminergic pathways, and enhancing leptin sensitivity [1]. They offer potential alternatives for patients intolerant to GLP-1 analogs.

#### 4.4.1. Cagrilintide

Cagrilintide is a long-acting AMY analog that acts as a dual agonist for both AMY and CTRs. A Phase 2 trial conducted at 57 sites across 10 countries showed that cagrilintide led to more significant weight loss than placebo and liraglutide, with reductions of up to 10.8% or 11.5 kg. In comparison, placebo resulted in a 3.0% weight loss, while liraglutide produced a 9.0% reduction (9.6 kg) [64]. Furthermore, combining cagrilintide with the GLP-1 receptor agonist semaglutide resulted in additional weight loss benefits. In a 32-week, multicenter, Phase 2 trial with 92 participants, the average weight loss at week 32 was more pronounced with CagriSema (semaglutide and cagrilintide) (15.6%) compared to semaglutide (5.1%) and cagrilintide (8.1%) [65]. The Phase 3 REDEFINE program^14^ is currently investigating CagriSema in individuals who are overweight and obese.

#### 4.4.2. Petrelintide (ZP8396)

Petrelintide is a long-acting, once-weekly, subcutaneous AMY analog and a potent dual AMY and calcitonin receptor agonist. It is stable at neutral pH with minimal precipitation, which enables potential coformulation with peptide compounds. A Phase 1 clinical trial showed its weight reduction effect, and a Phase 2b trial has commenced that is anticipated to achieve a similar decrease in body weight as GLP-1 receptor agonists yet with enhanced tolerance [66].

#### 4.4.3. Amycretin (NNC0487-0111)

Amycretin is a long-acting coagonist of GLP-1 and AMY and is available in formulations for once-daily oral and once-weekly subcutaneous injection administration. The Phase 1 trial of oral amycretin in obesity, involving 16 participants, showed a 13.1% reduction in weight after 12 weeks, outperforming current weight loss medications. The drug demonstrated a favorable safety profile and high tolerability, with adverse effects consistent with those observed in previous trials involving GLP-1 and CagriSema [67].

### 4.5. GLP-1/GLP-2 dual agonist: dapiglutide

Dapiglutide is a first-in-class dual GLP-1 and GLP-2 receptor agonist. Pharmacotherapy targeting GLP-2 was demonstrated to enhance epithelial barrier function in the small intestine. Dapiglutide alleviates comorbidities associated with low-grade inflammation by enhancing intestinal barrier function through GLP-2 and leverages the weight loss effects of a potent GLP-1 agonist. A Phase 1 trial demonstrated a mean weight loss of up to 4.5% in 4 weeks [68]. This data facilitated the progression of dapiglutide in clinical development for obesity. A Phase 2 study conducted over 12 weeks is investigating the effects of lower dapiglutide doses on body weight, while also assessing alterations in gut permeability and inflammation.^15^ GLP-2 is a gastrointestinal hormone known for intestinal growth-promoting and antiapoptotic effects. Rodent studies demonstrated that exogenous GLP-2 administration increases colon adenoma growth, suggesting a potential role in intestinal tumor progression. The tolerability of GLP-2 treatment for 30 months has only been examined; however, its safety with prolonged use remains uncertain. This raises concerns regarding the potential long-term risks of developing or progressing intestinal tumors with extended GLP-2 treatment, necessitating further research [69].

### 4.6. GIP agonism vs. antagonism: MariTide

As previously mentioned, several GLP-1 receptor agonists have shown efficacy in obesity management. While GLP-1 receptor agonists remain effective, there is an ongoing search for combination therapies to achieve higher and sustained weight loss and enhanced tolerability. Human genome-wide association studies indicated that the GIP receptor gene locus plays a role in regulating body weight, and GIP receptor knockout mice are resistant to obesity. In obese mice and monkeys, pharmacologically blocking the GIP receptor with anti-GIPR antibodies counteracted weight gain. Additionally, combining GIP receptor antagonism with GLP-1 receptor agonism synergistically decreased body weight [70]. One probable reason for the comparable impacts on weight loss seen with both GIP agonism and antagonism could be the potential desensitization of GIP receptors due to prolonged exposure to GIP agonists [71]. Another plausible explanation is that unlike GIP overexpression, which acts through central mechanisms, GIP antagonism via antibodies does not penetrate the blood-brain barrier. Instead, it exerts its effects peripherally, such as in the area of the postrema, which regulates food intake and provides protection against obesity [1,70]. MariTide (maridebart cafraglutide, previously known as AMG 133) is a bispecific molecule comprising a GIP receptor antagonist antibody conjugated to two GLP1 agonists. In a Phase 1 trial in adults with obesity, multiple ascending doses of MariTide 420 mg caused a weight decrease of 4.9% by day 7 and 14.5% by day 85, in contrast to minimal changes observed in the placebo group. The study participants maintained a weight loss of up to 11.2% 150 days after the last dose, highlighting sustained weight loss effects. The most common adverse events were mild nausea and vomiting, which were usually resolved within 48 h post-MariTide administration. It is undergoing evaluation in a Phase 2 trial [70].

### 4.7. Mitochondrial uncouplers

Mitochondrial uncoupling is the dissociation process between membrane potential generation and its utilization for ATP synthesis in the mitochondria [72]. Mitochondrial uncouplers transport protons through the inner membrane of mitochondria with a pathway that operates separately from ATP synthase. Uncoupling offers advantageous effects against lipid toxicity observed in patients with obesity, MAFLD, and MASH through an increase in fatty acid β-oxidation [38]. Mitochondrial uncoupling contributes to around 20–40% of energy expenditure and influences the basal metabolic rate. Importantly, weight loss with uncoupling results in adipose tissue loss with muscle mass preservation [73,74]. Historically, the most prominent example of a mitochondrial uncoupler used for obesity management is dinitrophenol. Nevertheless, serious adverse effects such as hyperthermia, cataract formation, and mortality led to its removal from the market by the FDA in 1938 [38]. Hyperthermia is a potentially lethal condition and occurs due to systemic mitochondrial uncoupling. Although toxicity concerns stalled the uncoupler’s therapeutic development in the early 1930s, new-generation liver-specific mitochondrial uncouplers have shown promise in treating metabolic diseases without serious adverse effects. Protonophores are agents that induce uncoupling [72], and controlled metabolic accelerators are molecules that leverage the natural uncoupling process [73].

#### 4.7.1. TLC-6740

TLC-6740 is a liver-specific mitochondrial protonophore that treats obesity and related conditions such as MASH. TLC-6740 increased energy expenditure by approximately 20% in obese mice models without affecting food intake [74]. Moreover, a Phase 1 trial conducted in healthy individuals confirmed the safety of TLC-6740 when administered over 10 days. Only mild adverse events were noted during that period. Pharmacokinetic data endorsed once-daily oral administration. Additionally, resting energy expenditure increased in a dose-dependent manner. This was accompanied by a decrease in the respiratory quotient, which is indicative of enhanced fat oxidation. There were also dose-dependent improvements in cholesterol levels and liver function tests [75].

#### 4.7.2. HU6

HU6 is a regulated metabolic accelerator that undergoes hepatic metabolism to produce the mitochondrial uncoupler 2,4-dinitrophenol. This process enhances substrate utilization, promoting the oxidation of fats and various carbon sources. In a Phase 2 trial, the average liver fat reduction at day 61 was 33.0% in the 450-mg group compared to a 5.4% increase in the placebo group. HU6 has shown potential as a promising pharmacological treatment for the management of obesity and its associated metabolic complications, such as liver steatosis [73].

### 4.8. NLRP-3 inhibitors: NT-0249 and NT-0796

Obesity is a chronic low-grade inflammatory disease. Systemic and cerebral inflammation plays a vital role in the pathogenesis of obesity and related metabolic diseases. Inflammasomes serve as intracellular sensors that regulate the secretion of inflammatory cytokines. The NLRP3 (NOD-, LRR- and pyrin domain-containing protein 3) inflammasome is a complex protein structure that forms in reaction to cellular disturbances [76]. This complex formation triggers caspase-1 activation, which facilitates the release of inflammatory cytokines interleukin-1β and interleukin-18 and induces inflammatory cell death, known as pyroptosis [77]. Saturated fatty acids abundant in obesity can potentially trigger NLRP3 inflammasome activation [76]. Thus, targeting NLRP3 could be a beneficial therapeutic strategy. Prior studies have demonstrated that NLRP3 knockout mice are resistant to diet-induced obesity [77]. Two different clinical-stage NLRP3 inhibitors, NT-0249 and NT-0796, have demonstrated that inhibiting NLRP3 reverses established diet-induced obesity in mice and provides metabolic benefits that extend beyond reversing obesity itself [76]. NT-0796 specifically targets immune cells contributing to inflammatory diseases, with therapeutic benefits in peripheral and neuroinflammatory diseases [78]. NT-0796 demonstrated a more notable impact on weight reduction than NT-0249 and exhibited efficacy similar to semaglutide. In contrast to semaglutide and calorie restriction, only NLRP3 inhibitors significantly lowered cardiovascular inflammatory markers like sVCAM-1 and PCSK9, which indicates potential benefit in cardiovascular risk reduction in individuals with obesity [76]. Additionally, weight regain after GLP-1 receptor agonist discontinuation may be decreased using a brain-penetrant NLRP3 inhibitor that supports weight loss maintenance [76,78].

### 4.9. MGAT-2 inhibitor: S-309309

Monoacylglycerol acyltransferase 2 (MGAT2) is an enzyme that facilitates dietary fat absorption through the resynthesis of triacylglycerol in the small intestine and regulates its assembly into chylomicrons for distribution to the liver [79,80]. Elevated MGAT2 expression in the liver leads to disrupted triglyceride homeostasis and the development of MAFLD/MASH [79]. The metabolic benefit of genetic deletion/inhibition of MGAT2 was evidenced in mice models with increased energy expenditure, decreased hepatic triglyceride levels, resistance to developing insulin resistance and obesity, and increased postprandial GLP-1 concentrations in response to a high-fat diet [79,80]. Therefore, targeting this pathway with MGAT2 inhibitors such as S-309309 could be an effective intervention strategy. A Phase 2 trial evaluated the safety and efficacy of S-309309, an oral once-daily MGAT2 inhibitor, in 365 individuals with obesity.^16^ The primary outcome was the percent body weight change at week 24. A release of the overall results is forthcoming.^16^

### 4.10. Gene therapies

Multiple anabolic pathways in adipogenesis serve physiological purposes for healthy fat tissue development. However, these pathways can become pathogenic in obesity, leading to overflowing, inflamed adipose tissue and insulin resistance, which contribute to weight gain and exacerbate obesity-related complications. Genome-wide association studies revealed hundreds of genes linked to obesity, representing a wide array of potential targets. The loss of function in these genes holds promise for protective effects against obesity. In line with these, targeting of GPR75 [81], Inhibin beta E (INHBE) [82], and activin receptor-like kinase 7 (ALK7) [83] via gene silencing technologies such as GalNAc-small interfering RNA (siRNA) or RNAi technology are currently being tested. The mechanism of ARO-INHBE involves decreasing INHBE gene expression in the liver and lowering the secretion of its product, namely activin E [82]. ARO-ALK7 works by diminishing the expression of ALK7, a receptor found in adipose tissue. Targeting of this pathway is anticipated to enhance lipolysis and decrease adipose dysfunction and visceral fat accumulation. Notably, it promotes the preservation of lean mass, enhancing overall body composition [83]. A clinical trial is scheduled for 2025 to evaluate WVE-007, which utilizes GalNAc siRNA to silence the INHBE gene [84].

## 5. Discussion

The alarming rise in obesity prevalence worldwide has broad consequences that not only affect the health of individuals but also significantly impact economies and overall society. On a personal level, it predisposes patients to many chronic diseases, including type 2 diabetes, cardiovascular disorders, pulmonary diseases, infertility, and various malignancies [85]. The obesity epidemic also exerts pressure on healthcare systems and economic resources, exacerbating social inequalities and worsening disparities in health outcomes. Tackling this public health issue requires a comprehensive approach integrating prevention and treatment strategies. Fortunately, the latest developments in the medical management of obesity are decreasing the discrepancy between pharmacological and surgical techniques, as new-generation antiobesity medications are achieving levels of weight loss once primarily associated with surgical interventions. This development marks a significant shift in the field and offers patients alternative avenues for substantial weight reduction. In addition, these GLP-1 analogs have therapeutic benefits in treating comorbidities. In people suffering from obesity, both with or without diabetes, they lead to major benefits in cardiovascular and renal outcomes [23,24,27,28]. Additionally, these medications offer substantial therapeutic benefits in managing obesity-associated conditions that often lack many effective treatments, such as HFpEF and OSA [30,31]. They significantly aid in weight loss. In addition, they effectively manage the entire package of obesity-related diseases by improving comorbidities. Moreover, these medications have much more tolerable side effect profiles, mainly gastrointestinal complaints, in comparison to older-generation antiobesity medications, such as the commonly used phentermine [1]. Given that obesity is a chronic condition necessitating long-term management, the availability of safe medications with minimal side effects is crucial. Numerous developing antiobesity medications are in the pipeline; whether they use recombinant peptides, small molecules, monoclonal antibodies, or RNAi technology, each has distinct mechanisms of action, including increasing energy expenditure and promoting muscle preservation. Some of these drugs, such as SRK-439 and NT-0249, are evaluated in preclinical studies [52,76]. Although animal data can be informative, one must use caution in interpreting these results for humans, as species differences in metabolism, energy expenditure, and brown adipose tissue content can lead to significant variability in observed effects [86].

## 5. Conclusion

Obesity is a complex disease with a multitude of comorbidities, contributing to a significant disease burden. The new era of weight management, driven by recent advancements, leverages diverse mechanisms of action to target various physiological pathways, resulting in decreased food intake, enhanced energy expenditure, preserved muscle mass, reversed liver steatosis, and improved tolerability in clinical trials. Successful translation of these trials into the clinical setting and ensuring equitable access to treatment options will be crucial, as they will potentially provide tolerable, effective, and sustainable solutions for weight management.

## Figures and Tables

**Table 1:** Current FDA-approved antiobesity medications.

**Phentermine/Topiramate**	Sympathomimetic/GABAergic
**Bupropion/Naltrexone**	NDRI/Opioid Antagonist
**Liraglutide**	Short-acting GLP-1 Analog
**Semaglutide**	Long-acting GLP-1 Analog
**Tirzepatide**	GIP/GLP-1 Agonist Chimeric Peptide
**Orlistat**	Lipase Inhibitor
**Setmelanotide**	MC4R Agonist

**Table 2:** Antiobesity medications in the clinical development pipeline.

**Phase 3 Clinical Trial**	Retatrutide (LY3437943)	GIP/GLP-1/Glucagon Agonist
**Phase 3 Clinical Trial**	Survodutide (BI 456906)	GLP-1/Glucagon Agonist
**Phase 3 Clinical Trial**	Mazdutide	GLP-1/Glucagon Agonist
**Phase 3 Clinical Trial**	Cagrilintide	Long-acting Amylin Analog
**Phase 3 Clinical Trial**	Orforglipron	GLP-1 Agonist
**Phase 3 Clinical Trial**	Taldefgrobep alfa (BHV-2000)	Myostatin Inhibitor
**Phase 2 Clinical Trial**	Bimagrumab	ActRII Inhibitor
**Phase 2 Clinical Trial**	Trevogrumab (REGN 1033)	Selective Myostatin Inhibitor
**Phase 2 Clinical Trial**	Garetosmab	Activin A Inhibitor
**Phase 2 Clinical Trial**	Pemvidutide	GLP-1/Glucagon Agonist
**Phase 2 Clinical Trial**	Petrelintide	Long-acting Amylin Analog
**Phase 2 Clinical Trial**	Dapiglutide	GLP-1/GLP-2 Agonist
**Phase 2 Clinical Trial**	MariTide	GLP-1 Agonist/GIP Antagonist
**Phase 2 Clinical Trial**	HU6	Controlled Metabolic Accelerator
**Phase 2 Clinical Trial**	NT-0796	NLRP3 Inhibitor
**Phase 2 Clinical Trial**	S-309309	MGAT2 Inhibitor
**Phase 2 Clinical Trial**	GSBR-1290	GLP-1 Agonist
**Phase 2 Clinical Trial**	VK2735	GLP-1/GIP Agonist
**Phase 1 Clinical Trial**	Amycretin (NNC0487-0111)	GLP-1/Amylin Agonist
**Phase 1 Clinical Trial**	TLC-6740	Liver-targeted Mitochondrial Protonophore
**Phase 1 Clinical Trial**	KER-065	Selective ActRII Ligand Trap
**Phase 1 Clinical Trial**	CT-996	GLP-1 Agonist
**Phase 1 Clinical Trial**	NT-0249	NLRP3 Inhibitor
**Preclinical Study**	RKER-034	ActRII Ligand Trap
**Preclinical Study**	SRK-439	Selective Myostatin Inhibitor
**Preclinical Study**	Anti-GPR75	Gene Silencing
**Preclinical Study**	WVE-007	Gene Silencing
**Preclinical Study**	ARO-INHBE	Gene Silencing
**Preclinical Study**	ARO-ALK7	Gene Silencing

## Footnotes

1 United States Food & Drug Administration (2023). Wegovy [online]. Website https://www.accessdata.fda.gov/drugsatfda_docs/label/2023/215256s007lbl.pdf [accessed 13 July 2024]

2 National Institutes of Health (2024). Semaglutide in NASH [online]. Website https://clinicaltrials.gov/study/NCT04822181?term=ESSENCE&intr=semaglutide&rank=2 [accessed 09 July 2024].

3 National Institutes of Health (2024). Search Results [online]. Website https://clinicaltrials.gov/search?cond=addiction&intr=semaglutide [accessed 09 July 2024].

4 National Institutes of Health (2024). SURMOUNT-MMO [online]. Website https://clinicaltrials.gov/study/NCT05556512?term=SURMOUNT-MMO%20&rank=1 [accessed 09 July 2024].

5 World Obesity Federation (2023). World Obesity Atlas [online]. Website https://s3-eu-west-1.amazonaws.com/wof-files/World_Obesity_Atlas_2023_Report.pdf [accessed 21 September 2024].

6 United States Food & Drug Administration (2024). Drug Shortages [online]. Website https://www.accessdata.fda.gov/scripts/drugshortages/dsp_ActiveIngredientDetails.cfm?AI=Semaglutide%20Injection&st=c [accessed 09 July 2024].

7 National Institutes of Health (2024). CT-996 [online]. Website https://clinicaltrials.gov/study/NCT05814107 [accessed 22 September 2024].

8 National Institutes of Health (2024). Bimagrumab and Semaglutide [online]. Website https://clinicaltrials.gov/study/NCT05616013?intr=bimagrumab&rank=1 [accessed 21 September 2024].

9 National Institutes of Health (2024). COURAGE [online]. Website https://clinicaltrials.gov/study/NCT06299098?intr=garetosmab&rank=3 [accessed 10 July 2024].

10 World Health Organization (2024). Clinical Trials Registry Platform [online]. Website https://trialsearch.who.int/Trial2.aspx?TrialID=ACTRN12623001233617 [accessed 22 September 2024].

11 National Institutes of Health (2024). TRIUMPH-3 [online]. Website https://clinicaltrials.gov/study/NCT05882045?term=TRIUMPH3%20&intr=Retatrutide&rank=1 [accessed 22 September 2024].

12 National Institutes of Health (2024). Survodutide [online]. Website https://clinicaltrials.gov/search?term=SYNCHRONIZE&intr=survodutide%20 [accessed 22 September 2024].

13 National Institutes of Health (2024). Pemvidutide [online]. Website https://clinicaltrials.gov/study/NCT05989711?intr=Pemvidutide%20&rank=1 [accessed 23 June 2024].

14 National Institutes of Health (2024). REDEFINE [online]. Website https://clinicaltrials.gov/search?term=REDEFINE%20&intr=Cagrilintide [accessed 24 September 2024].

15 National Institutes of Health (2024). DREAM [online]. Website https://clinicaltrials.gov/study/NCT05788601?intr=dapiglutide&rank=1 [accessed 06 July 2024].

16 National Institutes of Health (2024). S-309309 [online]. Website https://clinicaltrials.gov/study/NCT05925114 [accessed 27 June 2024].
